# Chromosome Genome Assembly and Annotation of the *Capitulum mitella* With PacBio and Hi-C Sequencing Data

**DOI:** 10.3389/fgene.2021.707546

**Published:** 2021-08-18

**Authors:** Duo Chen, Xuehai Zheng, Zhen Huang, Youqiang Chen, Ting Xue, Ke Li, Xiaozhen Rao, Gang Lin

**Affiliations:** The Public Service Platform for Industrialization Development Technology of Marine Biological Medicine and Products of the State Oceanic Administration, Fujian Key Laboratory of Special Marine Bioresource Sustainable Utilization, Southern Institute of Oceanography, College of Life Sciences, Fujian Normal University, Fuzhou, China

**Keywords:** *Capitulum mitella*, chromosome genome assembly, PacBio, Hi-C analysis, genome evolution analysis

## Introduction

A pedunculate barnacle *Capitulum mitella* (Linnaeus, 1758) is a dominant intertidal cirripede (Lee et al., 2000). *C. mitella* distribute in the mid- and high-intertidal zone of tropical and subtropical coasts and is common in the East Sea and South Sea of China. *C. mitella* has a great commercial value in China, the petiole muscle of which has been a source of traditional delicious seafood (Yuan et al., 2016). The local market demand led to high-intensity catching activity, and this behavior caused significant decrease in quantity and habitat destruction in recent years. Artificial aquaculture of fishery resources will help to reduce overexploitation of natural stocks and meet market needs for fishery products.

In general, the barnacle life cycle consists of six nauplius instars and a nonfeeding cyprid instar. In the barnacle life history, the cyprid is a transitory phase between the pelagic and sessile lifestyle. The cyprid role is responsible for locating, exploring, and attaching to a suitable substratum; subsequently, a complex metamorphosis and permanent settlement happen (Lagersson and Høeg, 2002). Cypris attachment and metamorphosis are also known as “cypris settlement” (Clare and Matsumura, 2000; Franco et al., 2016). The cypris settlement is a crucial event for the survival and development of both adults and subsequent generations. Within the last three decades, the basic biology of *C. mitella* such as reproductive characteristics, sperm ultrastructure, and larval culture conditions was investigated in our lab. *C. mitella* cyprids have been cultured on a relatively large scale. However, they cannot attach and metamorphose into juveniles in an artificial environment, so they would die eventually and interrupt their life history.

In recent years, the cypris morphology of *C. mitella* has been depicted minutely using SEM (Rao and Lin, 2014). After we had successfully induced metamorphosis of the *C. mitella* cyprids into juveniles, we described a time line and specific morphological changes during the metamorphosis under microscopy and SEM (Lin and Rao, 2017). The impacts of external (i.e., water temperature and salinity) and internal (i.e., cyprid age) factors on the metamorphosis of *C. mitella* were researched (Rao and Lin, 2020). These efforts are possible for completing the life history in an artificial environment and aquaculture of *C. mitella*. Not only is the artificial aquaculture for *C. mitella* a production activity but also it helps to reduce the stress on overexploited populations.

Barnacles are frequently dominant marine foulings and widely distributed around the world that play crucial roles in marine ecology (Maréchal and Hellio, 2011). Recently, there are a few transcriptome researches on barnacles, such as a model animal *Amphibalanus amphitrite* (Chen et al., 2011; Yan et al., 2012; Chandramouli et al., 2015; Sarah et al., 2016), a giant barnacle *Megabalanus volcano* (Yan et al., 2017) and a stalked barnacle *Neolepas marisindica* (Ryu et al., 2019). These efforts showed that the transcriptome databases could greatly promote the research on molecular mechanisms of barnacle settlement. Genome sequencing has been performed for an acorn barnacle *Amphibalanus amphitrite*, but there is still no genome sequencing information for a stalked barnacle. Numerous reports on *C. mitella* were mainly related to mitochondrial DNA sequencing and quantitative proteomics analyses (Song and Yoon, 2013; Yoon et al., 2013; Yuan et al., 2016; Tian et al., 2020). So far, no genome literature on the *C. mitella* is available. Whereas a lack of genome information on the barnacles has obstructed further inquiry on the molecular mechanisms with development, attachment, metamorphosis, genetic evolution, and so on, so does on *C. mitella*.

Accordingly, in this study, we aimed to sequence and assemble the genome of *C. mitella*. The baseline data obtained from this study will be very useful in taxonomical identification, phylogenetic analysis, larval settlement mechanisms, artificial breeding and aquaculture, and species protection for *C. mitella*.

## Materials and Methods

### Sample Preparation and Genome Sequencing

*C. mitella* specimens were sampled from the intertidal zone of Dinghai (26.2487°N, 119.7987°E), Fujian Province, China, and immediately frozen in liquid nitrogen. Total DNA was isolated from fresh muscle samples with the DNeasy® Tissue Kit (Qiagen, Hilden, Germany) according to the DNeasy® Protocol for animal tissues. Qubit 3.0 (Thermo Fisher Scientific, Inc., Carlsbad, CA, USA) was used to assess the DNA concentration and quality before downstream sequencing. Approximately 5 μg of high-quality genomic DNA was sheared and size-selected (~40 kb) for PacBio library construction followed by sequencing on the PacBio Sequel II platform (PacBio Biosciences, Menlo Park, CA, USA). Sequence libraries with inserts of average 350 bp were constructed for Illumina paired-end (PE) sequencing according to the manufacturer's instructions, and sequencing was subsequently performed on Illumina HiSeq X Ten platform (Illumina, San Diego, CA, USA).

### Genome Assembly

**Figure 1:**
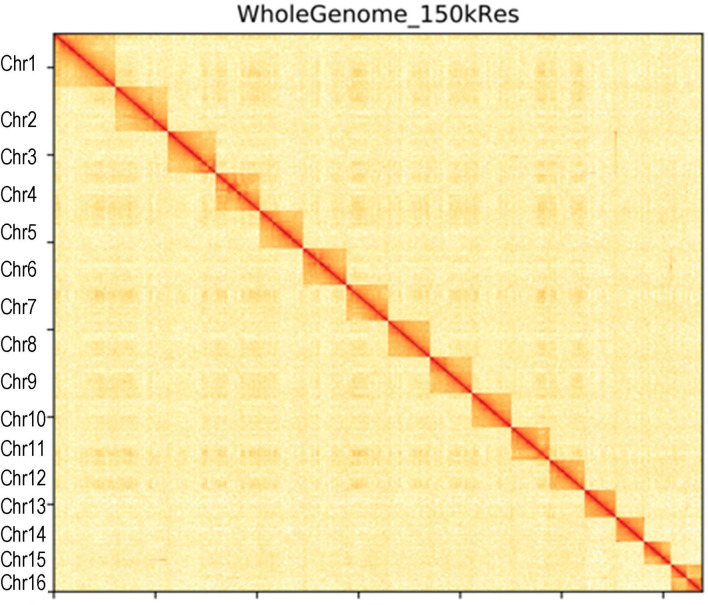
Hi-C interaction heatmap of *Capitulum mitella* genome. The heatmap shows that the genome was split into 100-kb bins, and the interaction links of individual chromosomes were validated.

**Figure 2:**
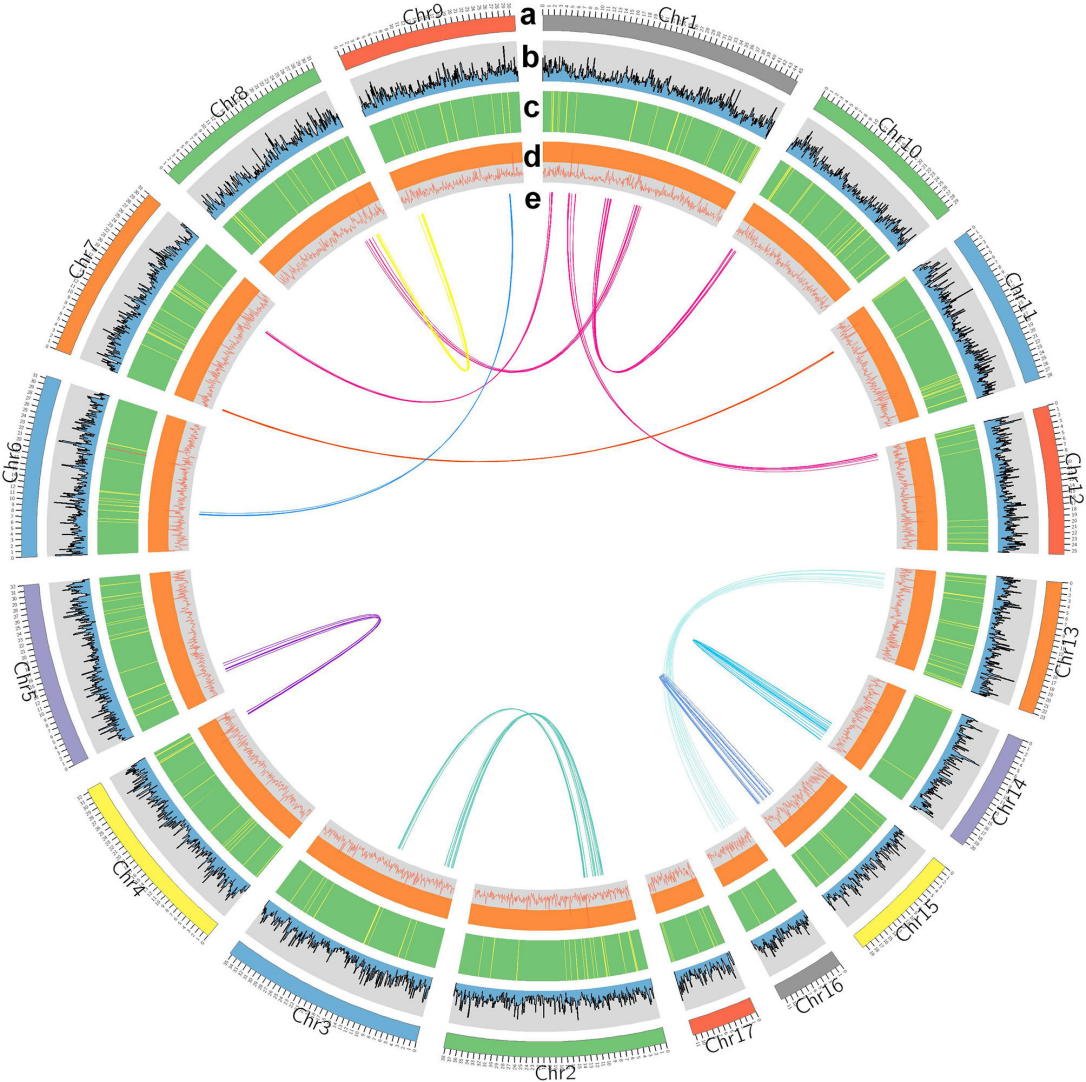
Characterization of *Capitulum mitella*. Genome elements are arranged in the Circos plot. **(a)** Eight assembled chromosomes of the genome. **(b)** The distribution of GC content. **(c)** Distribution of gene density (green). **(d)** Statistics for coverage of TEs. **(e)** Circos diagram of major interchromosomal relationships in the *C. mitella* genome. Each line represents a syntenic block; block size = 3 kb.

Canu (V2.0, https://github.com/marbl/canu/) (Koren et al., 2017) was used to perform the genome *de novo* assembly based on the PacBio long reads. After the initial assembly, primary contigs were corrected for three cycles with the Illumina reads using Pilon (V1.24, https://github.com/broadinstitute/pilon) (Belton et al., 2012). We used fresh muscle tissue from the same individual as in genome sequencing for the Hi-C library construction and sequencing. The Hi-C library was generated using Mbo I restriction enzyme following *in situ* ligation protocols (Peng et al., 2021). In brief, fresh muscle tissue was sampled and frozen in liquid nitrogen. Tissues were cross-linked with fresh formaldehyde and quenched with glycine. The total DNA was extracted from the nuclei in the lysis buffer [10 mM Tris-HCl (pH 8.0), 10 mM NaCl, 0.2% NP40, and complete protease inhibitors (Roche)]. The purified DNA was digested with Mbo I and was labeled by Biotin-14-dATP (Thermo Fisher Scientific) and then ligated by T4 DNA Ligase. After ligation, the cross-linking was incubated with proteinase K overnight; the ligated DNA was sheared into 200–600-bp fragments and then blunt-end repaired and A-tailed, followed by purification through biotin-streptavidin-mediated pull-down. Finally, the Hi-C libraries were quantified and sequenced on the Illumina HiSeq X Ten platform using a PE-150 module. The Hi-C sequencing data were then aligned to the scaffolds using BWA-MEM alignment algorithm (V1.2.2, https://github.com/lh3/bwa). Two ends of reads were independently aligned to the genome and only selected the read pairs for which both ends were uniquely aligned to the genome. The hiclib software (V0.5.7, http://github.com/hiclib) and a previously reported method (Gong et al., 2018) were applied to filter the Hi-C reads, and the interaction frequency was quantified and normalized among contigs. Lachesis (Burton et al., 2013) with default parameters was then applied to cluster contigs with the agglomerative hierarchical clustering method using the interaction matrix between sequences. To construct a high-quality assembly, we sequenced the genome by utilizing a combination of Illumina (~78.52 Gb of raw data), PacBio single-molecule real-time (SMRT) sequencing (~60.23 Gb of raw data), and Hi-C chromosome-scale scaffolding (~44.40 Gb of raw data) (Belton et al., 2012). The raw reads were filtered out by certain low-quality scrap reads and/or by minimum CLR length. After read quality filtering by PacBio Quality checking tool (http://github.com/PacificBiosciences/), a total of 60.17 Gb of long-read sequence data were produced, comprising 4,342,961 subreads with a read N50 of 19.979 Kb. This initial genome assembly was 463.09 Mb in length with a contig N50 of 3.14 Mb (Table 1; Figures 1, 2). A total of 259,360,062 reads (87.63 Gb) with a Q30 of 93.23% were generated for Hi-C analysis. Finally, 16 chromosomes anchored by the contigs were generated with the guidance of HiC reads; this result consists of the karyotype analysis (Supplementary Figure 1). This chromosome-level assembly has an average length of 28.11 Mb, with the shortest chromosome of 19.66 Mb (Chr16) and the longest one of 45.43 Mb (Chr1) (Figure 2). The scaffold N50 reached 3.14–31.03 Mb (Table 1), providing a chromosome-level genome assembly for *C. mitella*. The completeness of this assembled genome was assessed based on BUSCO analysis, which revealed that nearly 94.4% of the Arthropoda orthologs were included in the assembled (Table 2). These results indicated a high-quality assembly of *C. mitella* genome.

**Table 1:** Assembly statistics for *Capitulum mitella*.

**Items**	**Canu**		**Hi-C**	
	**Contig_len (Mb)**	**Contig_** **number**	**Scaffold_len (Mb)**	**Scaffold_** **number**
Total	463.09	1,462	482.98	269
Max	11.04	–	45.43	–
Number ≥2 kb	–	1,462	–	217
N50	3.14	–	31.03	–

**Table 2:** Assessment of the completeness of the *Capitulum mitella* genome assembly by BUSCO.

**Type**	**Number**	**Percent (%)**
Complete BUSCOs (C)	957	94.4
Complete and single-copy BUSCOs (S)	951	90.3
Complete and duplicated BUSCOs (D)	42	4.1
Fragmented BUSCOs (F)	15	1.5
Missing BUSCOs (M)	41	4.1
Total BUSCO groups searched	1,013	100

### Genome Annotation

The assembled *C. mitella* genome was homology-based annotated using a collection of protein-coding genes and RNA sequencing data from nine animal species: *Apis mellifera, Caenorhabditis elegans, Crassostrea gigas, Daphnia pulex, Drosophila melanogaster, Mizuhopecten yessoensis, Limulus polyphemus, Penaeus vannamei*, and *Plutella xylostella*. *D. pulex* and *P. vannamei* belong to subphylum Crustacea. *D. melanogaster, P. xylostella, and A. mellifera* belong to the subphylum Hexapoda, which is closely related to *C. mitella. M. yessoensis* and *C. gigas* belong to phylum Mollusca, and their ontogeny is similar to that of *C. mitella* through larval metamorphosis and attachment. *De novo* gene predictions were carried out by the AUGUSTUS package (V2.0) provided by BRAKER (V2.1.4) (Hoff et al., 2015). The MAKER pipeline was employed to identify the 21,899 protein-coding genes (Holt and Yandell, 2011), integrating protein sequences and transcript genes from the *de novo* assembly of *C. mitella* transcriptome data. All protein-coding genes were functionally annotated BLASTP (V2.2.3) (Anoop and Agostinho, 2015) against the public protein sequence databases EggNOG, Gene Ontology (GO), Cluster of Orthologous Groups (COG), and Kyoto Encyclopedia of Genes and Genomes (KEGG) with an E-value ≤ 1e−5. We functionally annotated 14,204, 9,312, 14,204, and 5,368 genes to EggNOG, GO, COG, and KEGG, respectively (Table 3). Small RNAs (sRNAs) and noncoding RNAs (ncRNAs) were predicted by Rfam and miRNA databases using tRNAsan-SE (V2.0) (Bernard et al., 2014) and BLASTN (V2.2.3), respectively. Some types of ncRNAs consisting of microRNAs (miRNAs) and small nuclear RNAs (snRNAs) were identified using INFERNAL (V.1.1.4) by searching the Rfam database (http://infernal.janelia.org/). In total, 136 miRNAs, 748 tRNAs, 56 rRNAs, and 87 snRNAs were identified in *C. mitella* genome (Supplementary Table 1). Tandem Repeats Finder (V4.09.1), LTR_FINDER (V1.07), and RepeatMasker (V4.0) (González and Deyholos, 2012) were combined to predict repeat sequences in the *C. mitella* genome. In the *C. mitella* genome, repetitive sequences accounted for 3.53%, and long terminal repeat (LTR) retrotransposons accounted for 0.28% of the genome, including 0.18% Ty3/gypsy, 0.03% Ty1/copia, and 0.06% other (Supplementary Table 2).

**Table 3:** General function annotation results statistics.

**Annotation statistics for nuclear genome**	**Number**	**Percent (%)**
Total protein	21,899	
EggNOG	16,224	74.08
GO	9,312	42.52
COG	14,204	64.86
KEGG	5,368	24.51
At least in one database	18,176	82.99

*COG, Cluster of Orthologous Groups; GO, Gene Ontology; KEGG, Kyoto Encyclopedia of Genes and Genomes*.

### Genome Evolution and Gene Family Analysis

**Figure 3:**
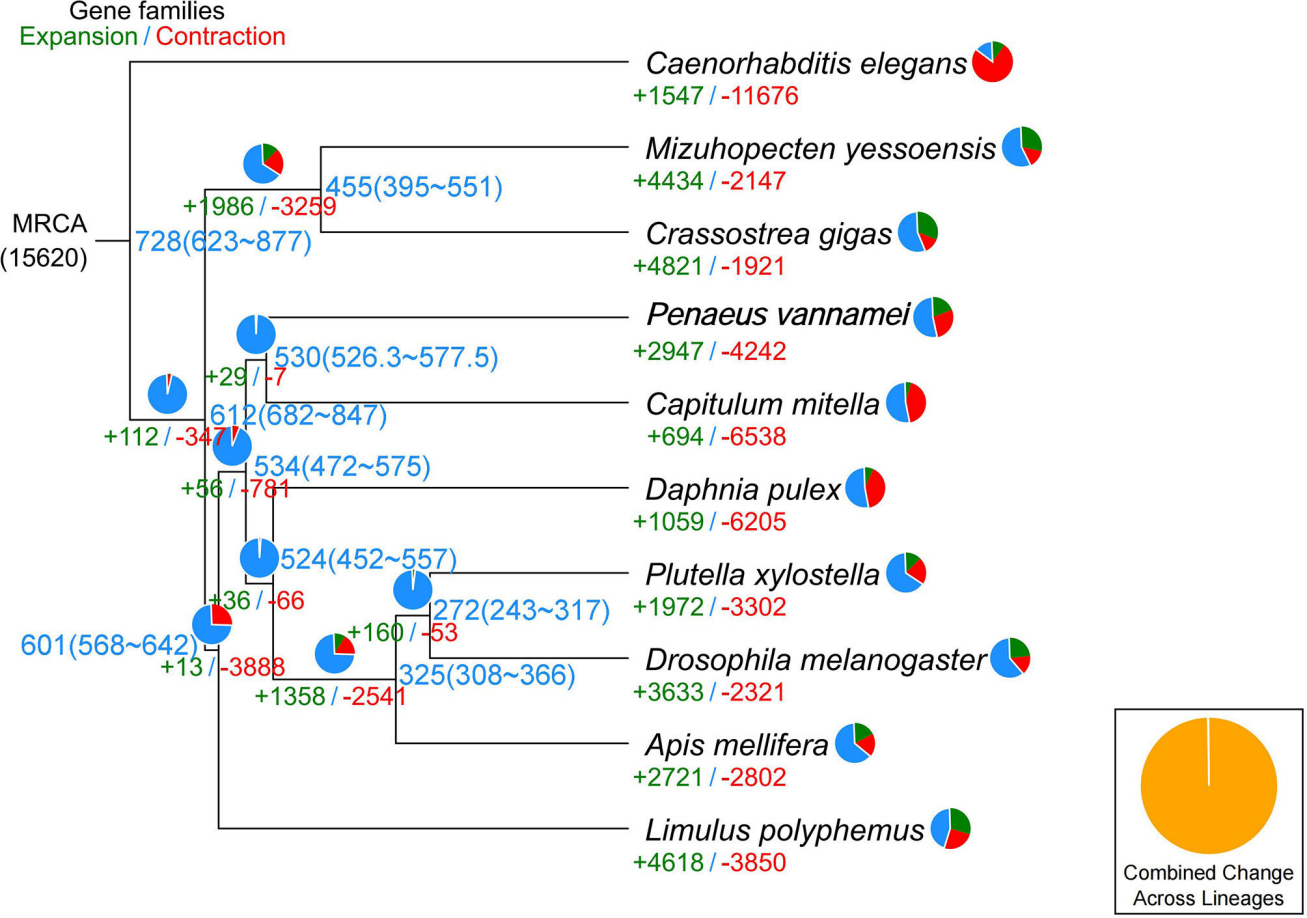
Phylogenetic tree showing the number of gene families displaying expansion (green) and contraction (red) among 10 animal species. The pie charts show the proportions of expanded (green), contracted (red), and conserved (blue) gene families among all gene families. The estimated divergence time (million years ago) is displayed in the phylogenetic tree in black. MRCA, most recent common ancestor.

**Figure 4:**
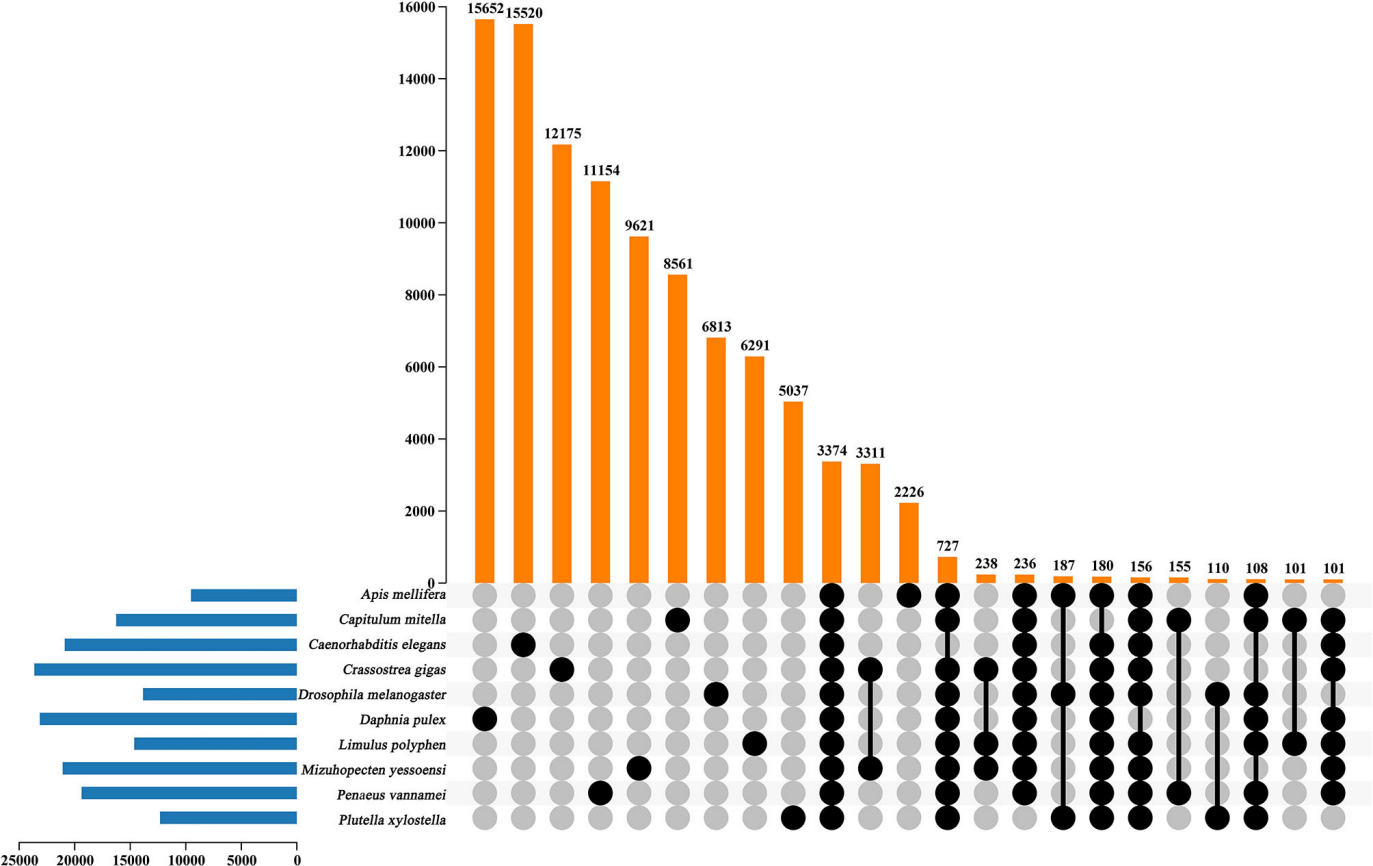
UpSet plot of the intersection of gene families in *A. mellifera, C.mitella, C. elegans, C. gigas, D.melanogaster, D. pulex, M. yessoenisis, L. polyphemus, P. vannamei and P. xylostella*. The numbers of gene families (clusters) are indicated for each species and species intersection.

In the current study, in order to investigate the evolution relationships among *C. mitella* and nine other species, we also perform phylogenomic analysis based on the amino acid sequences. These 10 species were *A. mellifera, C. mitella, C. elegans, C. gigas, D. melanogaster, D. pulex, M. yessoensis, L. polyphemus, P. vannamei*, and *P. xylostella*. Their protein sequences were acquired from the NCBI database, and single-copy genes were obtained by OrthoFinder (V2.27) (Emms and Kelly, 2015). Protein sequences of single-copy gene orthologs were aligned using MUSCLE software (V3.8.425). The alignments of Coding DNA Sequences (CDSs) that were guided by protein alignments were concatenated into the superalignment of nucleotide sequences. The phylogenetic relationships were then constructed using the maximum likelihood (ML) methods. On the basis of the protein alignments, the CDSs were aligned and then concatenated into a superalignment matrix for each family. The cluster size differences between the ancestor and each species were compared to analyze the expansion and contraction of the gene families using CAFE (V2.1) (Koichiro et al., 2007). The phylogenetic tree showed that *C. mitella* and *P. vannamei* phylogenetically were clustered into an independent ~530 million years ago (Mya), which was earlier than those of Insecta (325 Mya) (Figure 3). Comparative genomic analyses were performed among abovementioned 10 animal species, and we detected 15,620 families of homologous genes, among which 7,232 gene families were identified in *C. mitella*, including 694 and 6,538 gene families showing contraction and expansion, respectively (Figure 3). GO analysis revealed that the 694 expanded orthogroups were involved in the metabolic process, developmental process, stimulus response, transporter activity, and catalytic activity (Supplementary Table 3). The analysis of KEGG pathways manifested that most of the 694 expanded genes were annotated to the signaling molecules and interaction, cell growth and death, glycine, environmental adaptation, and serine and threonine metabolism (Supplementary Table 4). The *C. mitella* is a common species in middle tidal areas of intertidal zones, distributed in rock surfaces or crevices. Therefore, *C. mitella* have evolved a complex mechanism to adapt to dramatically changing environments such as large temperature differences and desiccation. So, the expanded orthogroups in *C. mitella* are related to the metabolic process, stimulus process, environmental adaptation, and so on. By contrast, the group of contracted gene families was related to lipid metabolism, phenylalanine metabolism, signal transduction, immune system, environmental adaptation, and cell growth and death (Supplementary Table 5). The GO terms annotation of the contracted genes showed that they were most related to the stimulus response, biological regulation, catalytic activity, growth, reproductive process, signaling, and developmental process (Supplementary Table 6). The contracted gene families in this process related to the development in the insect, suggesting that *C. mitella* and the other compared species share the core signal regulation network in the developmental process. The comparison of *A. mellifera, C. mitella, C. elegans, C. gigas, D. melanogaster, D. pulex, M. yessoensis, L. polyphemus, P. vannamei*, and *P. xylostella* revealed that 3,374 (28.26%) of the 11,935 *C. mitella* gene families were shared by the other nine species, whereas 8,561 gene families were unique to *C. mitella* (Figure 4). GO functional analysis revealed that these 8,561 unique families were enriched in the immune system process, developmental process, signaling, aging, stimulus response, and calcium signaling pathway (Supplementary Table 7), while KEGG annotation presented that most of the 8,561 unique families were gathered in signal transduction and cysteine and methionine metabolism (Supplementary Table 8).

## Data Availability Statement

The whole genome sequence data reported in this paper available in the Genome Warehouse in National Genomics Data Center, Beijing Institute of Genomics, Chinese Academy of Sciences, under accession number GWHBAVK00000000.1 that is publicly accessible at https://bigd.big.ac.cn/gwh. The complete sequences generated in this study was deposited to the NCBI, under the accession PRJNA725059.

## Author Contributions

DC, XZ, ZH, and TX contributed to genome assembly and annotations. TX, ZH, and GL contributed to manuscript preparation. KL, ZH, GL, XR, and YC contributed to sampling and sequencing. All authors contributed to the article and approved the submitted version.

## Conflict of Interest

The authors declare that the research was conducted in the absence of any commercial or financial relationships that could be construed as a potential conflict of interest.

## Publisher's Note

All claims expressed in this article are solely those of the authors and do not necessarily represent those of their affiliated organizations, or those of the publisher, the editors and the reviewers. Any product that may be evaluated in this article, or claim that may be made by its manufacturer, is not guaranteed or endorsed by the publisher.

## Supplementary Material

Supplementary Figure 1 The karyotype of *C. mitella*.

Supplementary Table 1 Repetitive element annotations in *C. mitella*.

Supplementary Table 2 Repetitive element annotations.

Supplementary Table 3 GO enrichment of genes in the expansion families in *C. mitella* genome.

Supplementary Table 4 KEGG enrichment of genes in the expansion families in *C. mitella* genome.

Supplementary Table 5 KEGG enrichment of genes in the contraction families in *C. mitella* genome.

Supplementary Table 6 GO enrichment of genes in the contraction families in *C. mitella* genome.

Supplementary Table 7 GO enrichment of genes that were specifically identified in *C. mitella* genome.

Supplementary Table 8 KEGG enrichment of genes that were specifically identified in *C. mitella* genome.
