# Supplementary figures and images for: Chromosome Genome Assembly and Annotation of the Capitulum mitella With PacBio and Hi-C Sequencing Data
Source: Front Genet. 2021 Aug 18;12:707546. doi: 10.3389/fgene.2021.707546 (PMC8416341; doi:10.3389/fgene.2021.707546)

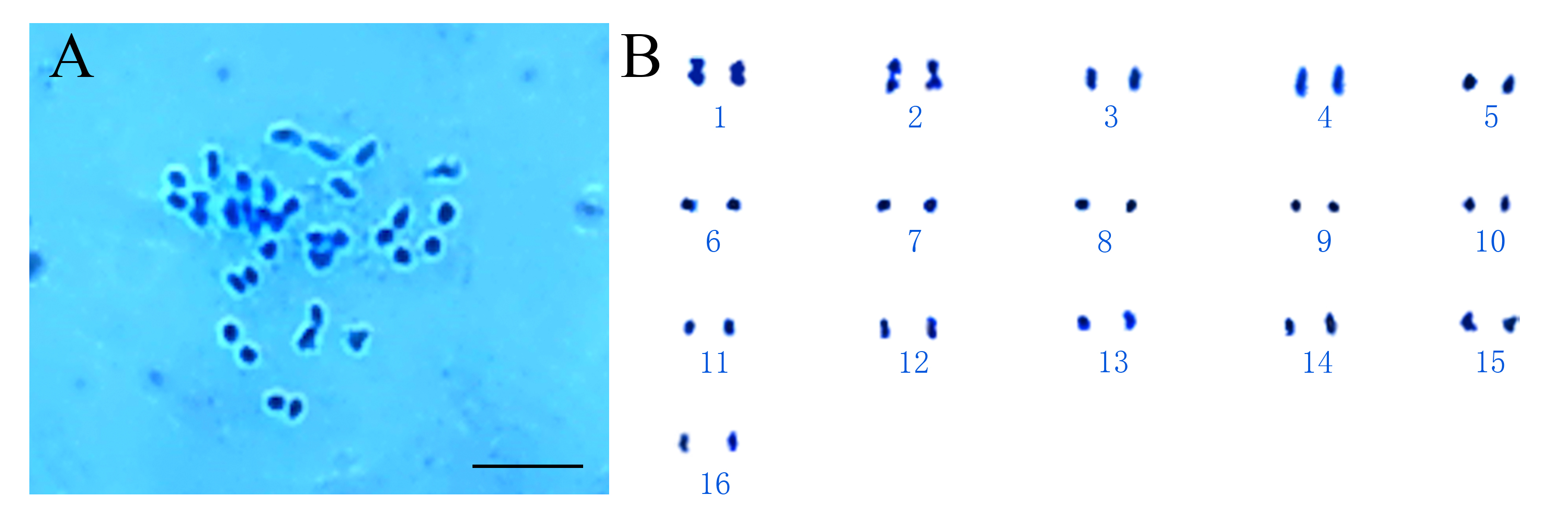

Supplement: Supplementary Figure 1 — The karyotype of C. mitella. [file Image_1.JPEG]
