# Supplementary material for: Chromosome Genome Assembly and Annotation of the Capitulum mitella With PacBio and Hi-C Sequencing Data
Source: Front Genet. 2021 Aug 18;12:707546. doi: 10.3389/fgene.2021.707546 (PMC8416341; doi:10.3389/fgene.2021.707546)
Supplement: Supplementary Table 1 — Repetitive element annotations in C. mitella. [file Table_1.DOCX]

Table S1 Repetitive element annotations in the *C. mitella.*

| Class | number | Total Len | Mean Len |
| --- | --- | --- | --- |
| Cis-reg; | 42 | 2587 | 61 |
| Gene; | 9 | 2682 | 298 |
| Gene; antisense; | 2 | 818 | 409 |
| Gene; lncRNA; | 2 | 256 | 128 |
| Gene; miRNA; | 136 | 13289 | 97 |
| Gene; rRNA; | 56 | 62795 | 1121 |
| Gene; ribozyme; | 3 | 838 | 279 |
| Gene; snRNA; snoRNA; CD-box; | 14 | 2286 | 163 |
| Gene; snRNA; snoRNA; HACA-box; | 5 | 684 | 136 |
| Gene; snRNA; snoRNA; scaRNA; | 1 | 129 | 129 |
| Gene; snRNA; splicing; | 67 | 10842 | 161 |
| Gene; tRNA; | 748 | 56159 | 75 |
