# Supplementary material for: Chromosome Genome Assembly and Annotation of the Capitulum mitella With PacBio and Hi-C Sequencing Data
Source: Front Genet. 2021 Aug 18;12:707546. doi: 10.3389/fgene.2021.707546 (PMC8416341; doi:10.3389/fgene.2021.707546)
Supplement: Supplementary Table 2 — Repetitive element annotations. [file Table_2.DOCX]

Table S2 Repetitive element annotations

|  | Number of elements* | Length occupied (bp) | Percentage of sequence |
| --- | --- | --- | --- |
| Retroelements | 20016 | 2696962 | 0.55% |
| SINEs | 251 | 14187 | 0.00% |
| Penelope | 1305 | 164391 | 0.03% |
| LINEs | 10903 | 1326903 | 0.27% |
| CRE/SLACS | 68 | 6960 | 0.00% |
| L2/CR1/Rex | 3651 | 376407 | 0.08% |
| R1/LOA/Jockey | 3137 | 504205 | 0.10% |
| R2/R4/NeSL | 178 | 30002 | 0.01% |
| RTE/Bov-B | 274 | 21099 | 0.00% |
| L1/CIN4 | 960 | 82744 | 0.02% |
| LTR elements | 8862 | 1355872 | 0.28% |
| BEL/Pao | 614 | 173543 | 0.04% |
| Ty1/Copia | 1267 | 152623 | 0.03% |
| Gypsy/DIRS1 | 5149 | 904412 | 0.18% |
| Retroviral | 1540 | 99526 | 0.02% |
| DNA transposons | 37529 | 3672883 | 0.75% |
| hobo-Activator | 8941 | 904120 | 0.18% |
| Tc1-IS630-Pogo | 5275 | 1077964 | 0.22% |
| En-Spm | 0 | 0 | 0.00% |
| MuDR-IS905 | 0 | 0 | 0.00% |
| PiggyBac | 175 | 16920 | 0.00% |
| Tourist/Harbinger | 1026 | 97586 | 0.02% |
| Other (Mirage,P-element, Transib) | 1010 | 59742 | 0.01% |
| Unclassified: | 2280 | 201626 | 0.04% |
| Total interspersed repeats: | 134330 | 6571471 | 1.33% |
| Small RNA: | 1121 | 147575 | 0.03% |
| Satellites: | 1498 | 218080 | 0.04% |
| Simple repeats: | 181472 | 10172922 | 2.07% |
| Low complexity: | 10564 | 606258 | 0.12% |
